# A Case of Transvaginal Small Bowel Evisceration following Hysterectomy with Discussion of Emergency Department Diagnosis and Management

**DOI:** 10.1155/2022/1334302

**Published:** 2022-02-02

**Authors:** Matthew Apicella, Maximiliano Mayrink, Chetan D. Rajadhyaksha, David A. Farcy

**Affiliations:** ^1^Department of Emergency Medicine, Mount Sinai Medical Center, Miami Beach, Florida, USA; ^2^Department of Obstetrics and Gynecology, Mount Sinai Medical Center, Miami Beach, Florida, USA; ^3^Department of Radiology, Mount Sinai Medical Center, Miami Beach, Florida, USA; ^4^Department of Emergency Medicine and Critical Care, Herbert Wertheim College of Medicine, Florida International University, Miami, Florida, USA

## Abstract

Transvaginal small bowel evisceration is a rare surgical emergency that requires urgent surgery to prevent bowel necrosis, sepsis, and death. It was first reported in 1864 by Hyernaux with less than 100 cases reported since the original publication. The overall mortality rate is reported as 5.6 percent. We present the case of a 49-year-old woman who presented to the emergency department with a chief complaint of moderate abdominal pain and vaginal bleeding for 1 hour. The patient reported that she underwent a robotic-assisted laparoscopic hysterectomy 11 weeks prior for uterine fibroids. Visual examination revealed a loop of the small bowel coming from the superior aspect of her vagina. Literature reviews have noted a higher incidence of dehiscence following robotic-assisted total laparoscopic hysterectomy. It is important for the emergency physician to make the diagnosis, initiate prompt consultation with departments of obstetrics and gynecology and general surgery, and treat for potential infection.

## 1. Introduction

Transvaginal small bowel evisceration is a rare surgical emergency that requires urgent surgery to prevent bowel necrosis, sepsis and death. It was first reported in 1864 by Hyernaux [1] with less than 100 cases reported since the original publication. Transvaginal small bowel evisceration is a rare complication following a hysterectomy that is associated with advance age, enterocele, and previous vaginal surgery. “Rates are hard to determine due to the rarity, but literatures shows a rate between 0.032% and 1.25” [2]. The overall mortality rate is reported as 5.6 percent; however, when the bowel is strangulated through the vagina, the defect morbidity is higher [3]. This case highlights the importance of early diagnosis, resuscitation of the patient, bowel preservation, rapid consultation, and definitive surgical repair. We report the case of a 49-year-old woman with small bowel evisceration after having a robotic-assisted laparoscopic hysterectomy.

## 2. Case Report

**Figure 1:**
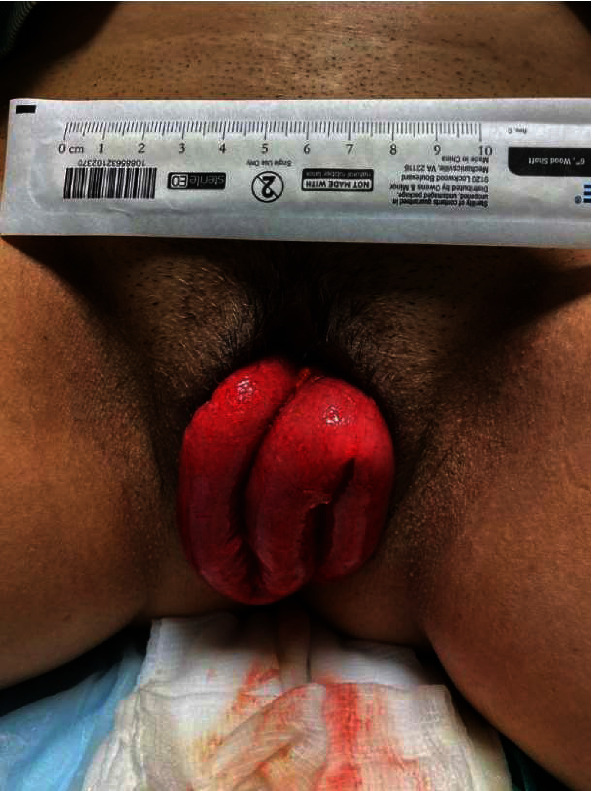
Loop of the small bowel protruding from the vagina that is hyperemic with no evidence of gross ischemia (photo with signed consent from patient).

**Figure 2:**
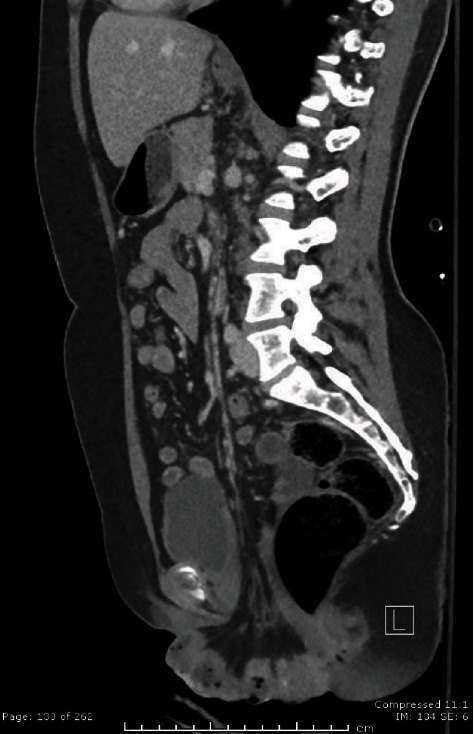
Axial CT image with intravenous contrast: image demonstrates pelvic floor dysfunction with rectovaginal space widening.

A 49-year-old woman G3P3 presented to the emergency department with a chief complaint of moderate abdominal pain and vaginal bleeding for 1 hour. The patient stated that while in the bathroom attempting to have a bowel movement, she “saw blood and felt something come out of her vagina.” Patient reported that she underwent a robotic-assisted laparoscopic hysterectomy 11 weeks prior for uterine fibroids. She followed all her postoperative instructions appropriately including one month of bed rest. She had no other past medical history. She did endorse increased pelvic pressure for the last few weeks when using the bathroom. She reported using a sex toy intravaginally 2 days prior to the incident but did not notice any pain or discomfort at the time. The patient described the location of her pain as bilateral lower abdominal. Although she was unable to describe the pain, she reported that it was constant and severe. She denied any fevers, nausea, vomiting, chills, diarrhea, or dysuria. She admitted to being able to pass gas. On initial presentation, her vitals were blood pressure of 104/65, pulse of 85 beats per minute, temperature of 37.7 °C, respiratory rate of 18 breaths per minute, and oxygen saturation of 97% on room air. On physical examination, she had moderate to severe tenderness to palpation in the suprapubic area without voluntary guarding, rebound, or rigidity. The patient was consented for a pelvic exam due to a new state law that went into effect on July 1, 2020, that mandates written consent for pelvic examination. Visual examination revealed a loop of the small bowel, hyperemic with no evidence of grossly ischemic areas coming from the superior aspect of her vagina (Figure 1); a full exam was deferred due to pain. We covered the loop of the bowel with moist dressing and consulted general surgery and gynecology. Since our department has a dedicated computed tomography (CT) scan, the patient underwent a CT of her abdomen and pelvis with IV contrast. While waiting for the consultants, the patient became more tachycardic, and after the review of her laboratory tests, the only abnormalities were WBC of 11.24 103/*μ*L (ref 4.810.80 103/*μ*L) with neutrophil 80.9% (ref 42.0-75.0%) and lactic acid of 2.8 mmol/L (ref 0.4-2.0 mmol/L). At this time, the patient was identified with severe sepsis and received broad-spectrum antibiotics with 4.5 g of piperacillin-tazobactam. Because of the COVID-19 virus, the patient had to await the result of her COVID-19 test before going to the operating room, per our hospital protocols. Computer tomography results revealed a pelvic floor dysfunction and rectovaginal space widening (Figure 2). Also, there was a large enterocele and wall thickening of the prolapsed small bowel with few foci of ectopic gas along the prolapsed bowel. No evidence of bowel obstruction was noted, but the presence of bowel ischemia or early bowel perforation could not be excluded by CT. The patient was taken for emergency surgery with both obstetrics and gynecology and general surgery. In the operating room, the patient was placed in dorsal lithotomy position, and under general anesthesia, a manual reduction of the eviscerated bowel was done. On speculum exam, complete vaginal cuff dehiscence was noted without any signs of infection. However, tissues between the anterior vaginal wall and the bladder wall were extremely friable and weakened. The granulation tissues at the edge of the vaginal cuff were removed, and the defect was closed in simple interrupted suture without any complications via vaginal approach. The patient received two days of cefoxitin postoperatively, and she was discharged without any complication. At three weeks postoperative follow-up, the patient is doing well without complication and without any abnormal vaginal bleeding.

## 3. Discussion

Vaginal cuff dehiscence following a hysterectomy is a rare occurrence with an estimated incidence of 0.032% and 1.25% [4]. The most notable chief complaint for this disease is acute abdominal pain with vaginal bleeding, vaginal protrusion, or vaginal discharge [4–6]. The median time reported for evisceration after a pelvic surgery is 20 months. Due to its rarity, the risk factors are limited to case reports and case report reviews of the literature; there is limited data on the risk factors for dehiscence following hysterectomy. A recent review of the literature noted a higher incidence of dehiscence following robotic-assisted total laparoscopic hysterectomy (2.33%) and total laparoscopic hysterectomy (0.87%). The lowest incidence noted was following total vaginal hysterectomy. Considering the surgical procedure, the patient underwent may assist in more appropriate triaging of patients with potentially higher level of acuity in the emergency department. The risk factors for a transvaginal small bowel evisceration vary when discussing postmenopausal and premenopausal women. The risk factors for premenopausal women consist of trauma caused by coitus, rape, or foreign bodies [1]. Advanced age, previous vaginal surgical intervention, enterocele repair, and sudden increase in intra-abdominal pressure such as cough or constipation are the risk factors for postmenopausal women [1]. A study conducted by Kowalski et al. found that 68% of the patients who developed evisceration were postmenopausal and of these, 73% had previous vaginal surgery and 63% had an enterocele [7]. These findings are consistent with our patient's presentation. Complications from transvaginal bowel herniation include bowel strangulation, bowel incarceration leading to ischemia, bowel perforation, intra-abdominal sepsis, peritonitis, septicemia, ileus, and deep vein thrombosis [1, 2, 5]. Diagnosis of evisceration is done by direct visualization during pelvic examination. This is not a radiological diagnosis and surgery should not be delayed, but if time permits, a CT scan of the abdomen and pelvis with IV contrast could assist the surgeons. Although not noted in our case, it also may be able to determine if an injury to the bladder can be seen before visualization in the operating room which would prompt earlier consultation to urology. In terms of emergency department management, the patient should be quickly assessed and resuscitated with fluid and started on broad-spectrum antibiotics to cover for gastrointestinal anaerobic bacteria [4]. Gynecologic and/or general surgery should be promptly notified because this is a surgical emergency and the definitive treatment is operative. The bowel should be kept moist with saline irrigation, and manual replacement through the vaginal cuff can be attempted if the bowel does not appear ischemic and the patient can tolerate it. If the reduction is successful, the patient will still need to be managed by surgery. If you are in an institution, where you have no general surgeon or obstetric/gynecologic surgeon, immediate transfer should be initiated.

## 4. Why Should an Emergency Medicine Physician Be Aware of This?

It is important for the emergency physician to make the diagnosis of transvaginal small bowl evisceration. Due to its high degree of morbidity and mortality, this diagnosis should not be confused with a rectal or uterus prolapse and a detailed examination must be done in a timely manner. They should initiate prompt consultation with departments of obstetrics and gynecology and general surgery, treat for potential infection, and initiate expedited transfer if these services are not available for definitive care. Although small bowel evisceration is a rare occurrence, it is important for the emergency physician to be aware of the possible complications patients who present posthysterectomy even if surgery was several years prior.
